# Spatial-temporal patterns of homicide in socioeconomically deprived settings: violence in Alagoas, Brazil, 2006‒2015

**DOI:** 10.1080/16549716.2021.1952752

**Published:** 2021-08-02

**Authors:** Kevan Guilherme Nóbrega Barbosa, Blake Byron Walker, Alessandra Vieira da Silva, Gessyca Luyze Procópio Gonzaga, Evanisa Helena Maio de Brum, Mara Cristina Ribeiro

**Affiliations:** a Mestrado Profissional Pesquisa Em Saúde, Centro Universitário Cesmac, Maceió, Alagoas, Brazil; b Institute for Geography, FAU Erlangen-Nürnberg, Erlangen, Germany

**Keywords:** Brazil, homicide, mortality, spatial-temporal analysis, violence

## Abstract

**Background:**

Homicide presents a significant health burden globally, but geographical differences in homicide rates necessitate focussed analyses of spatial and temporal patterns, particularly in affected areas. The highest rates are concentrated in regions in Central and South America, but analyses of sub-regional patterns and sex-specific differences may yield important information for addressing the upstream causes of homicide at the community level.

**Objective:**

This study examines and presents spatial and temporal patterns of homicide victims from 2006 to 2015 in the state of Alagoas, Brazil, focussing on the municipality scale and differentiated by victims’ sex.

**Methods:**

Data comprising victims’ age, sex, the date, time, and the municipality of the homicide incident were acquired from the Brazilian National Mortality Information System. These data were aggregated by municipality, and we made quantitative comparisons of sex-specific homicide rates between the capital city of Macieó metropolitan region and the peripheral, predominantly rural regions. Empirical Local Bayes methods were used to adjust per-capita homicide risk estimates and map the results.

**Results:**

A total of 19,560 homicides occurred during the study period, with an average of 60.4 per 100,000 inhabitants; the metropolitan region rate was 81.8, compared to 46.5 for the remaining regions. The male homicide rate was 115.9 per 100,000, compared to 7.1 for females. Empirical Local Bayes mapping showed strong clustering of male homicide risk in specific cities near the capital, while female risk was more dispersed throughout the region.

**Conclusions:**

The risk of male victim homicide observed for the metropolitan region of Alagoas was amongst the highest globally, particularly during the period 2012–2014. Geographical differences in male and female risk may indicate differences in risk factors and highlight a need for prevention programmes that take into account gender-specific pathways of violence.

## Background

In 2016, an estimated 477,000 persons were victims of homicide [1]. These external causes of death constitute a significant and avoidable global health burden, not only in terms of economic losses and life-years lost, but also for the psychosocial wellbeing of communities and individuals worldwide.

Legal definitions of homicide generally centre on the act of ending the life of another person, and doing so with malicious intent. While individual instances of homicide feature a unique set of circumstances, the literature identifies and traces common upstream pathways specifically highlighting the ways in which adverse social and material conditions can aggravate a person’s psychological state and thereby increase their probability of physically attacking another person [2]. In this way, homicide may be framed as reflecting adverse social, economic, and material contexts at the household or community level, with downstream effects negatively impacting on the psychosocial health of residents. This framing does not imply an environmental determinism of homicide, rather, it asserts that the socioeconomic condition of a given space plays an important role in psychosocial trajectories that can lead to violence.

Given the well-documented correlations between socioeconomic deprivation and homicide, geographical differences in homicide rates are observed throughout the literature. For example, the global distribution of homicide rates exhibits clusters in countries with high socioeconomic inequalities, such as in certain regions of Latin America [3]. This disparity is also observable at the sub-national/regional scale (e.g. state-level differences) and at local levels (e.g. differences between neighbourhoods) [4]. The use of geographical and temporal analyses therefore provide a useful means for visualising and heuristically examining homicide trends and patterns in order to inform and support appropriate upstream policy responses in affected areas.

The 2014 World Cup and 2016 Olympics brought international media attention to the high rates of violence in Brazil. Recent estimates from the Institute of Applied Economic Research (IPEA) indicated a clear upward trend since 2007, with a homicide rate of 31.6 per 100,000 inhabitants in 2017 [5]. By comparison, this is nearly 30 times the homicide rate in Germany and 158 times the homicide rate in Japan [6]. At the sub-national scale within Brazil, high homicide rates cluster in the relatively poor North-East region, with the state of Alagoas exhibiting amongst the highest homicide rates in the world being estimated at 71.4 per 100,000 inhabitants in 2011 [7]. During the last decade, homicide rates in Alagoas were over 50 per 100,000 inhabitants [7]. The interventions are urgently needed at state and national levels.

In addition to the geographical and socioeconomic dimensions of homicide, the literature indicates that sex and gender play significant roles [4]. The common pathways leading to homicide, the relationships between victim and perpetrator, locations/settings, and the mechanisms of homicide, all differ between male and female victims [8]. Male-victim homicide more commonly exhibits associations with other forms of criminality (e.g. linkages to organised crime), while female-victim homicides are often associated with familial- and/or intimate-partner violence. These differences present important considerations when analysing homicide and violence data, such that sex-specific (and where possible, gender-specific) patterns are taken into account.

The purpose of this study is to provide an overview of the sex-specific spatial and temporal patterns of homicide in Alagoas, Brazil. Of particular interest is the evolution of homicide rates and their differences by victim sex and geographical region. By mapping the distribution of reported homicides across the study area, we present an empirical basis for future policy evaluation and the targeting of prevention programmes. The understanding of geographical areas in most risk for homicide could help the governor to implement strategies to prevent this type of violence. The study’s hypothesis was that the homicides in the state were high around the metropolitan region, and that there was a pattern predominance of male victims in all municipalities of the state.

## Methods

### Study design and setting

The objective of this research is to examine the spatial and temporal patterns of homicide victims from 2006 to 2015 in the state of Alagoas, Brazil, focussing on the municipality scale and differentiated by victims’ sex. This study uses a geospatial approach based on descriptive-quantitative and cartographic analysis of administrative data for the ten-year period 2006 to 2015 inclusive. The study area comprises the entire state of Alagoas, located in North-Eastern Brazil. Alagoas contains 102 municipalities, including the capital city Maceió (2020 population 1.02 million). Alagoas is approximately the size of Massachusetts in the USA, has a total population of 3.35 million persons and features the third-highest population density in Brazil (approx. 112 people per km^2^) [9]. The state is located on the east coast of Brazil, where its famous beaches make the Alagoas coast a popular tourist destination and residential area for wealthy Brazilians. The concentration of wealth along the coast contrasts strongly against the extreme poverty and socioeconomic deprivation of Alagoas. The state has the highest illiteracy rate (17.2%) [10] and the second-lowest average income (approx. USD 145/month). The 2010 census data report a Gini index for household income of 0.63, indicating a high degree of polarity between rich and poor.

### Participants and data sources

Since 1975, the Brazilian Ministry of Health has monitored nationwide mortality through via the *National Mortality Information System* [11]. Deaths due to external causes, including the estimated date/time of death, the victim’s municipality of residence, and sociodemographic information about the victim, are reported in all states to the Federal Ministry of Health. This information is made available to the public via the *DATASUS* web system. We downloaded ten years of data (2006–2015), filtered by causes of death synonymous with homicide, based on the International Classification of Disease version 10 codes X85 to Y09 (ICD-10) [12], corresponding with the definition of homicide as ‘injuries inflicted by another person with intent to injure or kill, by any means’ [12]. Deaths related to law enforcement activities and deaths with unknown intent, were excluded.

Victims’ place of residence was also classified as being in the metropolitan region of Alagoas or in the inland region. The list of all municipalities, including estimated populations, are shown in Supplementary File 1. Additionally, the median monthly income of each municipality and the male and female populations were derived from the 2010 Brazilian National Census [13], selected as a proxy for socioeconomic status. Preliminary analyses indicated that median income had the most complete data and provided the most accurate representation of the socioeconomic geography of the study area.

### Variables

The study investigated six variables of interest such as: 1-homicide; 2-city of victims’ residence; 3-region of victims’ residence; 4-victims’ and family incomes for each city; 5-year of homicide occurrence; 6-sex of the victims. The first four variables were described in the previous item. The year of homicide range between 2006 and 2015 and the sex of the victim was considered female Vs male.

### Statistical methods

Sex-specific crude (i.e. non-adjusted) homicide rates per 100,000 residents were calculated for each year in the study period. The pooled 10-year crude homicide rates were computed using the 2010 sex-specific census populations. The study period was divided into three-year periods (2006–2008; 2009–2011; 2012–2014) to investigate trends in homicide rates over time. In order to identify municipalities with the highest homicide rates, those with a rate of more than 80 per 100,000 inhabitants were highlighted on the maps. All geographic data were analysed using the Brazilian standard SIRGAS 2000 polyconic projection in the ArcGIS software (v.10.4).

In order to reduce statistical instability and account for edge effects in the geospatial data (i.e. when cases occur near the boundary of a municipality and may be due to risk factors or correlates in the neighbouring municipality), the spatially-smoothed relative risk was computed, based on Empirical Local Bayes. This algorithm adjusts incidence rates based on the values of neighbouring areal units, creating a smoothing effect. This model was parameterised using a linear inverse-distance spatial weights matrix, where the maximum distance of influence between municipalities was set to 100 kilometres. The maximum distance was selected using Ripley’s K, an iterative algorithm for measuring spatial clustering and dispersion in geographical data, which demonstrates at which scale the strongest clustering effects are observed. Additionally, these results were validated using heuristic visual interpretation and sensitivity analyses run by the study authors who reside in the study area and are familiar with the geographical configuration of the space being analysed. Empirical Bayes was selected as it provides a non-parametric smoothing method to improve relative risk estimates based on local mean homicide rates. This adjustment process preserves the overall geographical distribution of homicide rates while reducing local variance, e.g. due to small numbers of observations in rural/remote regions and non-normal/non-Poisson data and parameter distributions [14]. The total number of homicides, crude rates, and relative risks were mapped for male and female victims separately using this approach. All geostatistical analyses were conducted using the GeoDA software (version 14.0).

The socioeconomic analysis used data from the Brazilian Census year 2010 to compare and search for correlations with homicide rates between 2009 and 2011. Other time periods were excluded to reduce bias, as incomes in the region may have changed between census periods. Results for all 102 cities are shown in Supplementary File 2. Ordinary least squares regression was then conducted to estimate associations between income and homicide rate in the study area. A second regression was undertaken with using a log-transformed income variable. Model fit and diagnostics were heuristically assessed.

### Ethics statement

This analysis was performed using publicly available secondary data accessed via DATASUS [15]. All analyses were conducted at the aggregated municipal scale or coarser, and individual victims are not represented. Data can be freely accessed on http://tabnet.datasus.gov.br/cgi/deftohtm.exe?sim/cnv/ext10AL.def

## Results

Over the ten-year study period 19,560 homicides were observed. The state-wide homicide rate from 2006 to 2015 was 60.4 per 100,000 inhabitants; 81.8 for the metropolitan region and 46.5 for the inland region. Male victims accounted for over 90% of all recorded cases, as shown in Table 1.

**Table 1.** Homicide deaths by sex and region in Alagoas state 2006–2015

		Year of Homicide Death									
		2006	2007	2008	2009	2010	2011	2012	2013	2014	2015
**Homicides**	*State*	1618 (100.0%)	1826 (100.0%)	1887 (100.0%)	1873 (100.0%)	2087 (100.0%)	2243 (100.0%)	2046 (100.0%)	2148 (100.0%)	2085 (100.0%)	1747 (100.0%)
	male	1512 (93.4%)	1718 (94.1%)	1804 (95.6%)	1762 (94.1%)	1950 (93.4%)	2105 (93.8%)	1913 (93.5%)	2005 (93.3%)	1960 (94.0%)	1652 (94.6%)
	female	106 (6.6%)	108 (5.9%)	82 (4.4%)	111 (5.9%)	137 (6.6%)	139 (6.2%)	133 (6.5%)	142 (6.6%)	125 (6.0%)	95 (5.4%)
	*Metro Region*	956 (100.0%)	1031 (100.0%)	1124 (100.0%)	989 (100.0%)	1134 (100.0%)	1194 (100.0%)	1057 (100.0%)	1159 (100.0%)	1000 (100.0%)	765 (100.0%)
	male	908 (95.0%)	989 (95.9%)	1078 (95.9%)	939 (94.9%)	1065 (93.9%)	1124 (94.1%)	990 (93.7%)	1092 (94.2%)	940 (94.0%)	714 (93.3%)
	female	48 (5.0%)	42 (4.1%)	46 (4.1%)	50 (5.1%)	69 (6.1%)	70 (5.9%)	67 (6.3%)	66 (5.7%)	60 (6.0%)	51 (6.7%)
	*Inner Cities*	662 (100.0%)	795 (100.0%)	763 (100.0%)	884 (100.0%)	953 (100.0%)	1049 (100.0%)	989 (100.0%)	989 (100.0%)	1085 (100.0%)	982 (100.0%)
	male	604 (91.2%)	729 (91.7%)	726 (95.2%)	823 (93.1%)	885 (92.9%)	981 (93.5%)	923 (93.3%)	913 (92.3%)	1020 (94.0%)	938 (95.5%)
	female	58 (8.8%)	66 (8.3%)	36 (4.7%)	61 (6.9%)	68 (7.1%)	69 (6.6%)	66 (6.7%)	76 (7.7%)	65 (6.0%)	44 (4.5%)

Male-victim homicide rates were consistently observed to be above 140 per 100,000 inhabitants, while female-victim homicide rates were observed to be lower than 10, as shown in Table 2.

**Table 2.** Homicide death rates by sex and region in Alagoas state 2006–2015

		Death rates per 100,000 inhabitants										
		2006	2007	2008	2009	2010	2011	2012	2013	2014	2015	Mean
**Homicides**	*State*	51.9	58.0	59.4	58.4	64.6	68.9	62.4	65.1	62.8	52.3	60.4
	male	98.7	110.2	115.7	112.1	123.2	132.2	119.4	124.5	121.1	101.6	115.9
	female	6.7	6.7	5.1	6.8	8.3	8.4	7.9	8.4	7.3	5.8	7.1
	*Metro Region*	79.3	84.2	90.6	78.7	89.2	92.9	81.4	88.5	75.7	57.4	81.8
	male	156.2	165.7	180.6	155.5	174.7	182.6	159.5	174.6	149.2	112.6	161.1
	female	7.7	6.6	7.1	7.7	10.4	10.5	9.9	9.6	8.7	8.0	8.6
	*Inner Cities*	34.6	41.3	39.4	45.3	48.6	53.2	49.9	49.7	54.3	48.9	46.5
	male	63.5	75.7	75.4	85.0	91.0	100.4	94.1	92.7	103.2	94.6	87.6
	female	6.0	6.8	3.7	6.2	6.9	6.9	6.6	7.6	6.4	4.4	6.2

**Figure 1.**
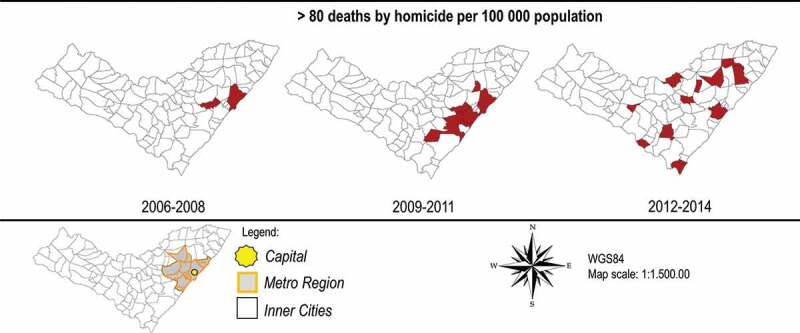
Municipalities with homicide rates >80 per 100,000

Figure 1 shows the temporal change in homicide rates for municipalities with exceptionally high incidence (>80). The number of municipalities with high rates increases, and after the emergence of a hotspot in the metropolitan area (2009–2011) there is dispersion into surrounding areas around the metropolitan area.

**Figure 2.**
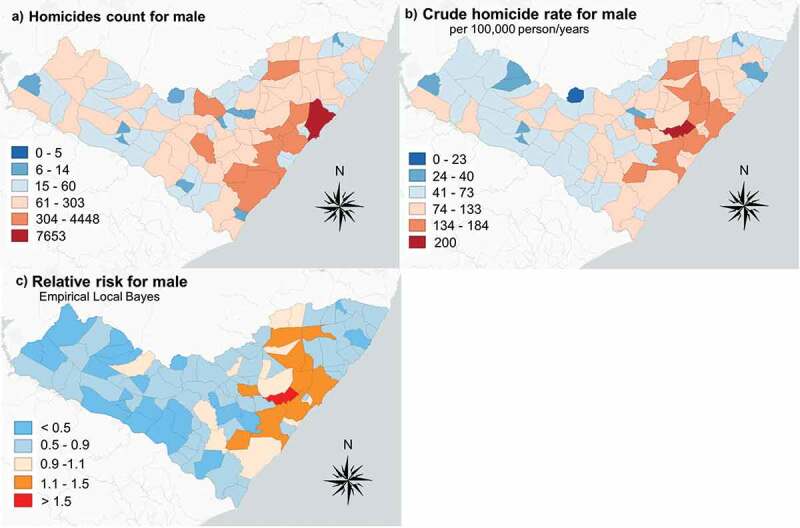
Empirical Bayes relative risk estimates for male-victim homicide

**Figure 3.**
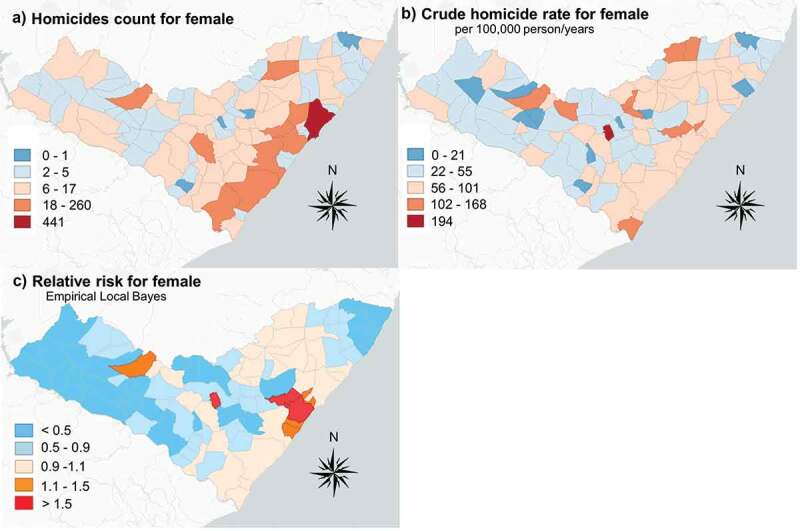
Empirical Bayes relative risk estimates for female-victim homicide

The geographical distributions of male and female homicides are similar, as shown in Figures 2 and Figures 3. However, disproportionately high rates were observed in the eastern part of Alagoas, most notably around the state capital of Maceió. The Empirical Bayes estimates indicate that the highest risk for males is concentrated in the city of Pilar, while the highest risk for females is found in the neighbouring municipalities of Pilar, Marechal Deodoro, and Coité do Nóia. Low rates and low relative risk estimates were observed for both male and female populations in the predominantly rural western half of Alagoas.

The socioeconomic analysis, shown in Table 3, indicates a positive correlation between income and the crude homicide rate, with an average increase of 10 homicides per 100,000 inhabitants for every increase of R$ 100 in average monthly household income (R$ = Brazilian currency; R$ 100 is approximately 20 USD USD or 16€ EUR). Average income accounted for slightly more than 30% of the variance in homicide rates in the study area. Model diagnostics indicated acceptable homoscedasticity and near-normality of residuals, indicating suitable model fit. The model featuring log-transformed income values exhibited slightly higher normality of residues and accounted for slightly less variance in homicide rates.

**Table 3.** Model results indicating positive correlation between income and homicide

	Not Transformed				Transformed			
Predictors	Estimates	Std. Error	Statistic	p	Estimates	Std. Error	Statistic	p
(Intercept)	−11.83	8.40	−1.41	0.162	−427.520	71.240	−6.00	<0.001
INCOME	0.10	0.02	6.67	<0.001				
INCOME_log					75.06	11.38	6.60	<0.001
Observations	102				101			
R^2/^R^2^ adjusted	0.308/0.301				0.305/0.298			

## Discussion

Our findings highlight trends and patterns of homicide in the socioeconomically deprived state of Alagoas, Brazil. We analysed 19,560 homicides from 2006 to 2015, or an average of 5.4 deaths per day. International comparisons place Alagoas amongst the most violent regions globally, with rates similar to those observed in Honduras, El-Salvador, Venezuela, and Colombia [16]. While these four countries have recently experienced civil war and other forms of violent social-civil disruption, the situation in Alagoas differs. In Brazil, the dramatic increase in homicide rates began in the 1980s, following a consolidation of organised crime coincident with an increase in poverty, particularly in urban areas [17]. This pattern is reflected in the geographic distribution of homicide, such that rates in the metropolitan region are nearly double those of the inland municipalities, demarcating a distinct urbanisation and dispersal of homicide rates during the study period. The metropolitan area of the state comprises 13 municipalities with one-third of the state’s population. Since 1999, homicide rates have been increasing in the metropolitan area, which has been linked to easy access and low cost of unregistered firearms, increases in drug trafficking in suburban areas, rapid urban population growth, increasing income inequality, and low rates of school attendance [18]. It is noteworthy that firearm use constitutes the most common mechanism of homicide in the study area, with links to organised crime, which include extensive acquisition and distribution networks for illicit firearms.

Similar to national studies of homicide, we observed that approximately nine in ten homicide victims were male, providing further evidence of the potential pathways leading to homicide in this region, such as disproportionately high rates of participation in violent organized crime [19]. According to official federal data [3], 91.8% of homicide cases between 2007 and 2017 in Brazil featured a male victim. Previous studies have examined the cultural associations between constructions and images of masculinity and firearms, such that firearms appear prominently in socioeconomically deprived and otherwise marginalised communities as a symbol of male dominance [20].

This study was unable to analyse homicides by perpetrator characteristics, as these data are not made available in the public database (and in many cases, the perpetrator remains unidentified). Previous studies using public security data indicate that the vast majority of perpetrators are male, and that the majority of homicides are men killing men, both of whom are usually young, poor, and black, with minimal access to formal education and a history of participation in organized crime [8,19–22].

In the Figure 1 we observed a distinctive geographic trend during the study period, such that, in the first transition period (2009–2011), the highest rates shifted from the metropolitan area (Maceió and Pilar) to the South-Eastern coast. The second transition period (2012–2014) exhibits an outward shift to the surrounding municipalities on the margins of the metropolitan area. This geographic transition of homicides has been coined Northeastern Brazil’s ‘internalization of violence’, referring to a general inland shift or expansion out of core metropolitan areas, such as Maceió, Recife, Natal, and Salvador [23,24]. The Empirical Bayes analysis highlighted the highest concentrations of homicide risk in the municipality of Pilar. This relatively small, suburban territory was home to 33,305 inhabitants in 2010 and is located approximately 30 kilometers (45 minutes by car) north-west of Macieó’s urban core. This observed pattern of dispersion, or suburbanization of violence, may be explained by organised crime dynamics in recent decades. Official data were unattainable for this study, but it is public knowledge that two large criminal factions, the First Capital Command (PCC) and Red Command (CV), have been locked in violent territorial dispute for domination of the drug market (primarily powder and crack cocaine) in Alagoas [25]. The PCC emerged in 1993 in the São Paulo prison system and has since spread to nearly all Brazilian states [26], whilst the latter emerged in the streets of Rio de Janeiro and benefits from close connections with militant organizations in the coca trade, predominantly with elements of the Revolutionary Armed Forces in Colombia (FARC) [27].

Female-victim homicide also featured cluster in Pilar, but also exhibited additional high-risk clusters in Marechal Deodoro and Coité do Nóia. While Marechal Deodoro is similar to Pilar in population, size, and structure, Coité de Nóia is a small rural municipality with approximately 10,000 residents, located 20 kilometers north of the city of Arapiraca. Femicide often features different pathways than male-victim homicide, such that the perpetrator is often a male (former) intimate partner or family member and the incident dynamics often include elements of physical and/or sexual dominance [28–30]. Recent literature is marked by a shift towards definitions of femicide that recognise the gendered dimensionality of female-victim homicide and reflects these elements accordingly; Diana Russel’s definition of femicide includes the killing of girls and women by men whose motivation stems from the victim’s gender, the psychological dynamic of which exhibits hatred, contempt, pleasure, and/or a sense of ownership [30,31]. The aforementioned psychosocial factors leading to femicide may be exacerbated by larger-scale socioeconomic conditions such as income inequality, poor infrastructure and living conditions, unstable family structures, lower levels of policing, and higher rates of youth involvement in criminal activity [8].

Compared to national-level data and other regions of Brazil, Alagoas and its neighbouring state Pernambuco have registered higher rates of femicide. While the Brazilian rate in 2013 was around 4.6 per 100,000 inhabitants [7], and the rate in the Northeast region of the country was 5.6 [32], the rate in Alagoas was markedly higher at 8.4. In the present investigation, the femicide peak was observed for the period 2010–2013, coincident with the highest rates of male-victim violence.

There are few studies examining risk factors for violence and homicide that focus on the Alagoas context. The most comprehensive investigation published to date is a report by the Secretary of Security of Alagoas, in which the authors mapped homicides from 2016–2020 and observed spatial correlations between male-victim homicide and absence of primary school, low reported income per households, and divorced persons. Similarly, Walker et al. [8] used exploratory statistical modelling and interviews with imprisoned youth and their families to identify neighbourhood covariates of male- and female-victim homicides. They concluded that indicators of low-quality or informal housing also demonstrate a significant correlation with homicide and can be linked to rapid increases in neighbourhood-scale socioeconomic inequality since the 1990s.

Similar to the studies mentioned above, we also observed that an increase in average monthly income was positively correlated with homicide rates. There high concentrations of household wealth in the state capital and the metropolitan region (the wealthiest in the state) and the region also has the highest homicide rates. Wealthy urban neighbourhoods often feature a lucrative market for powder cocaine, while socioeconomically marginalised urban neighbourhoods are often home to many crack cocaine users, making urban centres a high-value territory and therefore the sites of territorial conflict between criminal factions. In Brazil’s cities, wealthy and impoverished communities often coexist side-by-side, which can obfuscate poverty in datasets that use average income as a socioeconomic indicator.

One of the first national policies in Brazil specifically seeking to reduce the prevalence and impact of homicide was the National Policy for Reduction of Mortality by External Causes, passed in 2001 [33]. This was hailed as a successful measure, because the national average homicide rate dropped throughout the 2000s. In 2003, Law No. 10,826 [34] approved by the Lula government, increased penalties for firearm possession and this was followed by drops in homicide rates from 2003 to 2006 [35]. However, beginning in 2007 homicides increased again, and the last official national estimates showed a clear trend of increasing violence from 2007 to 2017 [3]. In 2019, President Jair Bolsonaro attempted to pass a series of decrees to deregulate gun possession and increase civilian firearm ownership, yet these policy initiatives were denied by the national Senate and Supreme Court. One of the proposed policies was eventually passed (Decree 9847), and this increased civilian access to firearms and authorized shooting classes for adolescents [36]. The matter of firearm policy remains a highly contested political discourse and public debate.

Other policy efforts have been put forth to specifically target violence against women. In 2006, Law No. 11,340 came into effect, by which mechanisms to curb domestic and family violence against women were instituted [37]. In 2013, Law No. 12,845 was enacted, ordering that all female victims of violence must be eligible for comprehensive care in all public hospitals in Brazil [38], and in 2015, Law No. 13,104 added femicide as a distinct category of felony in the Brazilian Penal Code, thereby increasing the prescribed penalties in cases involving domestic/family violence or disparagement or discrimination against women [39].

In order to achieve reductions in homicide rates, several lessons can be learned from successful initiatives in other regions, such as the USA Homicide Reduction Program. Since 2002, the National Violent Death Reporting System has provided comprehensive nationwide data on reported violence, including homicides [40]. Such surveillance programs are crucial for the structured analysis of patterns, including spatial, temporal, and sociodemographic factors that often yield direct policy relevance [41]. From 1993 to 2014, the USA experienced a 53% reduction in homicide. In addition to national surveillance and data-sharing co-operation between states, this success can be attributed to evidence-based prevention programs, improved policing strategies, and increases in data quality, consistency, and analytical techniques [42].

A large, nationwide spatial analysis of homicide data in Brazil (5,562 municipalities) found strong geographical clustering of violence in and around cities with a high degree of socioeconomic inequality [43]. Another study performed in the neighboring state of Pernambuco found that homicides are clustered in the metropolitan region of the capital Recife. Similar to our results this highlighted the importance of the Human Development Index indicators in building a prevention strategy [44]. A similar study located in the nearby state of Bahia observed a clear spatial expansion of homicides outward from the major urban centres, this being nearly congruent with our observations in Alagoas [45].

In our analysis, we observed a 16.7% decrease in homicide rates 2014 to 2015 in Alagoas. Whether this is part of a longer-term trend, cannot be determined with any degree of certainty due to data gaps. While estimated homicide rates from 2016 onwards can be accessed via the cited IPEA, these data are not available at a sufficient spatial resolution to allow for municipal-scale analysis. As the 2020 Brazilian Census has been significantly delayed by the COVID-19 pandemic, it is likely that the socioeconomic data necessary to longitudinally analyse this correlation will not be likely for several years. However, the observed reduction of homicide rates in 2015 is coincident with the new state government’s measures to strengthen public security, primarily through increased investment in law enforcement [46]. However, these measures likely experienced a lag effect, if any, and without detailed data, it is not possible to confirm whether increased spending in law enforcement explains the 2015 decrease in homicide. We echo previous studies in asserting that upstream measures to target socioeconomic inequalities and other related drivers of violence are necessary [8].

### Strengths and limitations

This study was the first to examine municipal-level trends of homicide in Alagoas, and identified a distinct dispersion pattern, an observation made possible due to the availability of sex-specific homicide data on a fine spatial scale. This important to identify areas in risk and where the problem is concentrated to intervene. In addition to providing information about the broader regional-scale patterns, we identified several key municipalities for further investigation; the next study phase will use qualitative methods to further examine the situations in Pilar, Marechal Deodoro, and Coité do Nóia.

There are limitations to this study. The rates indicated here are not adjusted for age structure or other confounders, and the gap from 2015 to the present day prevents analysis of the post-2015 reduction and potential correlates or causal factors. Additionally, within-municipality differences in homicide, e.g. between town centres and their rural peripheries, occur at a finer scale than the data used in this study. Aggregation of homicide data is however necessary in order to help protect the identities of the victims, and the authors do not call for the publication of more precise victim data. Additionally, this study only used average monthly income as a proxy for socioeconomic status, as preliminary modelling demonstrated that it was the most stable predictor for which complete data for all municipalities in Alagoas were available. Other studies have clearly demonstrated that additional socioeconomic factors play a demonstrable role in homicide risk [22]. Future statistical analyses should take these into account.

## Conclusions

Over the course of one decade, the homicide rate in the Brazilian State of Alagoas rose strongly, exhibiting a clear pattern of spatial dispersal from the metropolitan core of the Alagoas capital city Maceió into its surrounding, predominantly residential municipalities. This finding contributes further evidence of what previous studies have coined an ‘internalization of violence’, meaning that hotspots are geographically dispersing in recent years. We observed that male-victim homicides were concentrated along the Atlantic coast, while female-victim homicides were more dispersed. These differences may be explained by differential, gender-specific pathways of violence and necessitate further research to identify risk factors in the context of northeast Brazil.

## Supplementary Material

Supplemental Material

## Data Availability

The datasets generated and/or analysed during the current study are available in the DATASUS website [http://tabnet.datasus.gov.br/cgi/deftohtm.exe?sim/cnv/ext10AL.def].

## Paper context

This detailed ten-year geographic analysis of homicidal risk in the state of Alagoas, Brazil advances understanding of homicidal violence in the region. The study’s novel contribution was the analysis of a detailed spatial pattern which shows evidence of major social problems aligned with high socioeconomic inequalities.

## References

[1] World Health Organization. World Health Statistics 2018: monitoring health for the SDGs, sustainable development goals. WHO Press [Internet]. 2018. [cited 2019 Dec 8]. Available from: https://www.who.int/gho/publications/world_health_statistics/2018/en/

[2] Lachaud J, Donnelly PD, Denry D, et al. A population-based study of homicide deaths in Ontario, Canada using linked death records. Int J Equity Health. 2017;16:1–11. 28738872 10.1186/s12939-017-0632-9 PMC5525348

[3] World Health Organization. Global status report on violence prevention 2014. WHO Press [Internet]. 2014. [cited 2019 Dec 8]. Available from: https://www.who.int/violence_injury_prevention/violence/status_report/2014/en/

[4] Barbosa K, Walker BB, Schuurman N, et al. Epidemiological and spatial characteristics of interpersonal physical violence in a Brazilian city: a comparative study of violent injury hotspots in familial versus non-familial settings. PLOS ONE. 2019;14:1–19. 10.1371/journal.pone.0208304 PMC6322764 30615621

[5] Instituto de Pesquisa Econômica Aplicada. Atlas da Violência. 2019. [Internet]. 2019. [cited 2019 Dec 8]. Available from: http://www.ipea.gov.br/atlasviolencia/download/19/atlas-da-violencia–2019

[6] United Nations Office on Drugs and Crime. Summary of the global study on homicide 2019. [Internet]. 2020. [cited 2020 Apr 9]. Available from: https://dataunodc.un.org/GSH_app

[7] Instituto de Pesquisa Econômica Aplicada. Atlas da Violência. 2018. [Internet]. 2018. [cited 2019 Dec 8]. Accessed 08 Dec 2019. Available from: http://www.ipea.gov.br/portal/index.php?option=com_content&view=article&id=33410&Itemid=432

[8] Walker BB, Moura de Souza C, Pedroso E, et al. Towards a situated spatial epidemiology of violence: a placially-informed geospatial analysis of homicide in Alagoas, Brazil. Int J Environ Res Public Health. 2020;17:1–15. 10.3390/ijerph17249283 PMC7764635 33322481

[9] Departamento de Informática do SUS. População Residente. Estudo de estimativas populacionais por município, idade e sexo 2000-2020-Brasil. [Internet]; 2021. [cited 2021 Jun 21]. Available from: http://tabnet.datasus.gov.br/cgi/deftohtm.exe?popsvs/cnv/popbr.def

[10] Pesquisa Nacional por Amostra de Domicílios Contínua. 2018. [Internet]. 2019. Available from: https://www.ibge.gov.br/estatisticas/sociais/populacao/9171-pesquisa-nacional-por-amostra-de-domicilios-continua-mensal.html?=&t=downloads

[11] Morais RM, Costa AL. Uma avaliação dos sistema de informações sobre mortalidade. Saúde Debate. 2017;41:101–117.

[12] International classification of disease and related health problems 10th revision. Version 2016. [Internet]. 2019. [cited 2019 Dec 9]. Available from: https://icd.who.int/browse10/2016/en

[13] Instituto Brasileiro de Geografia e Estatística. 2020. [cited 2020 Jul 24]. [Internet]. Available from: https://censo2010.ibge.gov.br/resultados.html

[14] Casella G. An introduction to empirical bayes data analysis. Am Stat. 1985;39:83–87.

[15] Departamento de Informática do SUS. Deaths from external causes. [Internet]. 2019. [cited 2019 Dec 9]. Available from: http://tabnet.datasus.gov.br/cgi/deftohtm.exe?sim/cnv/ext10AL.def

[16] World Health Organization. Homicide: WHO global health estimates. 2020. [cited 2020 Apr 24]. 2015. [Internet]. Available from: https://apps.who.int/violence-info/homicide/

[17] Souza ER. [Homicides in Brazil: the Major Villain for Public Health in the 1980s]. Ciênc Saúde Colet. 1994;10:S45–60. Portuguese.

[18] Nascimento EO. [Acumulação social da violência e sujeição criminal em Alagoas]. Soc estado. Portuguese. 2017 May-Aug;32:465–485.

[19] Murray J, Cerqueira DR, Kahn T. Crime and violence in Brazil: systematic review of time trends, prevalence rates and risk factors. Aggression Violent Behav. 2013;18:471–483. 10.1016/j.avb.2013.07.003 PMC3763365 24027422

[20] Souza ER. [Masculinity and violence in Brazil: contributes to reflection in health field]. Ciênc Saúde Colet. 2005 Mar;10:59–70. Portuguese.

[21] Moura de Souza C. Violência letal e tráfico de drogas em Maceió. Rev Port Ciênc Crim. 2016;26:485–498.

[22] Ursin M. “Crack ends it all?” A Study of the interrelations between crack cocaine, social environments, social relations, crime, and homicide among poor, young men in Urban Brazil. Contemp Drug Probl. 2014;41:171–199.

[23] Andrade LT, Diniz AM. Spatial reorganization of homicides in Brazil and the interiorization thesis. R Bras Est Pop. 2013;30:S171–91.

[24] Soares Filho AM, Merchan-Hamann E, Vasconcelos CH. Expansion, displacement and ruralization of homicide in Brazil, between 2000 and 2015: a spatial analysis. Ciênc Saúde Colet. 2020;25:3097–3105. 10.1590/1413-81232020258.32612018 32785545

[25] Globo. G1 Alagoas News. Homicides in Pilar, AL, related to faction dispute for traffic, says police. 2018. [cited 2020 May 3]. [Internet]. Available from: https://g1.globo.com/al/alagoas/noticia/homicidios-no-pilar-al-tem-relacao-com-disputa-de-faccoes-pelo-trafico-diz-policia.ghtml

[26] Manso BP, Dias CN. PCC, Sistema prisional e gestão do novo mundo do crime no Brasil. Rev Bras Segur Pública. 2017;11:10–29.

[27] Misse M. Organized crime and common crime in Rio de Janeiro: affinities and differences. Rev Sociol Polit. 2011;19:13–25.

[28] Gomes IS. [Femicides: a long debate]. Rev Estud Fem. 2018;26:1–16. Portuguese.

[29] Meneghel SN, Hirakata VN. Femicides: female homicide in Brazil. Rev Saúde Pública. 2011;45:564–574. 21552758 10.1590/s0034-89102011000300015

[30] Meneghel SN, Portella AP. Femicides: concepts, types and scenarios. Ciênc Saúde Colet. 2017;22:3077–3086. Portuguese. 10.1590/1413-81232017229.11412017 28954158

[31] Consejo Centroamericano de Procuradores de Derechos Humanos. I informe regional: situación y análisis del femicidio en la región Centroamericana. San José: Mundo Gráfico S.A; 2006. 274 p.

[32] Waiselfisz JJ, da Violência M 2015: homicídio de mulheres no Brasil. 2015. [cited 2020 Apr 3]. [Internet]. Available from: http://flacso.org.br/?project=mapa-da-violencia.

[33] Brazilian Ministry of Health. Política nacional de redução da morbimortalidade por acidentes e violência. (Ordinance No. 737).May 16, 2001.

[34] Brazilian Presidency of the Republic. Provides for registration, possession and sale of firearms and ammunition, the National Weapons System - Sinarm, defines crimes and makes other provisions. (Law No. 10 826). Dec 22, 2003.

[35] Waiselfisz JJ Map of Homicide: the new patterns of homicide violence in Brazil. 2012. [cited 2020 Apr 19]. [Internet]. Available from: http://www.mapadaviolencia.net.br/pdf2012/mapa2012_web.pdfAccessed19April2020

[36] Brazilian Presidency of the Republic. Regulates Law No. 10 826, of December 22, 2003, to provide acquisition, registration, possession and sale of firearms and ammunition and the National Weapons System and the Military Management System of Arms. 2019. (Decree No. 9 847) Jun 25, 2019.

[37] Brazilian Presidency of the Republic. It creates mechanisms to curb domestic and family violence against women, under the terms of § 8 of art. 226 of the Federal Constitution, the Convention on the Elimination of All Forms of Discrimination against Women, and the Inter-American Convention to Prevent, Punish and Eradicate Violence Against Women; provides for the creation of Courts for Domestic and Family Violence against Women; amends the Code of Criminal Procedure, the Penal Code and the Law of Penal Execution; and makes other arrangements. (Law No. 11 340). 2006 Aug 7.

[38] Brazilian Presidency of the Republic. Provides the mandatory and comprehensive care for people in situations of sexual violence. (Law No. 12 845). 2013 Aug 1.

[39] Brazilian Presidency of the Republic. Amends art. 121 of Decree-Law No. 2,848, of December 7, 1940 - Penal Code, to provide for femicide as a qualifying circumstance for the crime of homicide, and art. 1 of Law 8,072, of July 25, 1990, to include femicide in the list of heinous crimes. (Law No. 13 104). 2015 Mar 9.

[40] Steenkamp M, Frazier L, Lipskiy N, et al. The national violent death reporting system: an exciting new tool for public health surveillance. Inj Prev. 2006;12:S3–5. 10.1136/ip.2006.012518 PMC2563479 17170168

[41] National Center for Injury Prevention. Division of Violence Prevention. The National Violent Death Reporting System (NVDRS): A powerful tool for prevention. 2020. [cited 2020 Jul 24]. [Internet]. Available from: https://www.cdc.gov/violenceprevention/pdf/nvdrs_overview-a.pdf

[42] Eisner M, Nivette A, Murray AL, et al. Achieving population-level violence declines: implications of the international crime drop for prevention programming. J Public Health Policy. 2016;37:66–80. 27638243 10.1057/s41271-016-0004-5

[43] Ingram MC, Costa MM, Spatial A. Analysis of homicide across Brazil’s municipalities. Homicides Studies. 2017;21:1–24.

[44] Silva C, Melo S, Santos A, et al. Spatial modeling for homicide rates estimation in pernambuco state-Brazil. Int J Geo-Inf. 2020;9:1–19.

[45] Souza TO, Pinto LW, Souza ER. Spatial study of homicide rates in the state of Bahia, Brazil, 1996-2010. Rev Saúde Pública. 2014;48:468–477. 25119942 10.1590/S0034-8910.2014048005201 PMC4203071

[46] Governor of Alagoas. Secretary of State for Public Security. In Alagoas, investment in public security exceeded R$ 77 million this year. 2019. [cited 2020 May 19]. [Internet]. Available from: http://seguranca.al.gov.br/noticia/2019/12/23/em-alagoas-investimento-na-seguranca-publica-ultrapassou-r-77-milhoes-este-ano/

