# Supplementary material for: Spatial-temporal patterns of homicide in socioeconomically deprived settings: violence in Alagoas, Brazil, 2006‒2015
Source: Glob Health Action. 2021 Aug 2;14(1):1952752. doi: 10.1080/16549716.2021.1952752 (PMC8330714; doi:10.1080/16549716.2021.1952752)
Supplement: Supplemental Material [file ZGHA_A_1952752_SM0888.zip › Supplementary/Supplementary file_1.docx]

| Supplementary material 1. Male and female population around the 102 cities of the Alagoas state. | | | | | | | | | | | | | | | | | | | | | | | | |  |
| --- | --- | --- | --- | --- | --- | --- | --- | --- | --- | --- | --- | --- | --- | --- | --- | --- | --- | --- | --- | --- | --- | --- | --- | --- | --- |
| **CENSUS CODE** | **CITY** | | **REGION** | | **MALE POPULATION** | | | | | | | | | | | | | | | | | | | |  |
|  |  | |  | | **2006** | | **2007** | | **2008** | | **2009** | | **2010** | | **2011** | | **2012** | | **2013** | | **2014** | | **2015** | |  |
| 2700102 | ÁGUA BRANCA | | INNER CITY | | 9908 | | 9962 | | 10005 | | 10047 | | 10083 | | 10112 | | 10321 | | 10352 | | 10382 | | 10302 | |  |
| 2700201 | ANADIA | | INNER CITY | | 9082 | | 9040 | | 9008 | | 8969 | | 8934 | | 8934 | | 8913 | | 8891 | | 8856 | | 8778 | |  |
| 2700300 | ARAPIRACA | | INNER CITY | | 102090 | | 103381 | | 104579 | | 105689 | | 106704 | | 107642 | | 108533 | | 109243 | | 109891 | | 110587 | |  |
| 2700409 | ATALAIA | | METRO REGION | | 22590 | | 22757 | | 22918 | | 23062 | | 23197 | | 23304 | | 23412 | | 23500 | | 23586 | | 23660 | |  |
| 2700508 | BARRA DE SANTO ANTÔNIO | | METRO REGION | | 6931 | | 7063 | | 7184 | | 7298 | | 7407 | | 7496 | | 7589 | | 7669 | | 7738 | | 7808 | |  |
| 2700607 | BARRA DE SÃO MIGUEL | | METRO REGION | | 3721 | | 3785 | | 3846 | | 3901 | | 3954 | | 3999 | | 4053 | | 4105 | | 4152 | | 4202 | |  |
| 2700706 | BATALHA | | INNER CITY | | 8358 | | 8474 | | 8586 | | 8685 | | 8773 | | 8858 | | 8907 | | 8986 | | 9054 | | 9115 | |  |
| 2700805 | BELÉM | | INNER CITY | | 2545 | | 2486 | | 2427 | | 2372 | | 2324 | | 2271 | | 2372 | | 2333 | | 2296 | | 2258 | |  |
| 2700904 | BELO MONTE | | INNER CITY | | 3642 | | 3657 | | 3670 | | 3682 | | 3691 | | 3717 | | 3444 | | 3444 | | 3472 | | 3490 | |  |
| 2701001 | BOCA DA MATA | | INNER CITY | | 13115 | | 13185 | | 13243 | | 13298 | | 13348 | | 13382 | | 13409 | | 13433 | | 13467 | | 13541 | |  |
| 2701100 | BRANQUINHA | | INNER CITY | | 5777 | | 5727 | | 5689 | | 5654 | | 5621 | | 5576 | | 5544 | | 5510 | | 5485 | | 5453 | |  |
| 2701209 | CACIMBINHAS | | INNER CITY | | 5107 | | 5154 | | 5192 | | 5230 | | 5261 | | 5299 | | 5337 | | 5360 | | 5393 | | 5423 | |  |
| 2701308 | CAJUEIRO | | INNER CITY | | 10209 | | 10293 | | 10373 | | 10443 | | 10521 | | 10590 | | 10598 | | 10642 | | 10552 | | 10591 | |  |
| 2701357 | CAMPESTRE | | INNER CITY | | 3352 | | 3372 | | 3398 | | 3415 | | 3430 | | 3441 | | 3454 | | 3458 | | 3471 | | 3479 | |  |
| 2701407 | CAMPO ALEGRE | | INNER CITY | | 24267 | | 24782 | | 25267 | | 25734 | | 26170 | | 26574 | | 26950 | | 27442 | | 27761 | | 28062 | |  |
| 2701506 | CAMPO GRANDE | | INNER CITY | | 4624 | | 4626 | | 4630 | | 4632 | | 4640 | | 4638 | | 4799 | | 4800 | | 4803 | | 4819 | |  |
| 2701605 | CANAPI | | INNER CITY | | 9011 | | 9024 | | 9030 | | 9041 | | 9050 | | 9059 | | 9083 | | 9104 | | 9159 | | 9177 | |  |
| 2701704 | CAPELA | | INNER CITY | | 9119 | | 9036 | | 8963 | | 8888 | | 8815 | | 8733 | | 8606 | | 8549 | | 8689 | | 8626 | |  |
| 2701803 | CARNEIROS | | INNER CITY | | 3946 | | 4022 | | 4097 | | 4162 | | 4229 | | 4300 | | 4360 | | 4298 | | 4342 | | 4382 | |  |
| 2701902 | CHÃ PRETA | | INNER CITY | | 3804 | | 3805 | | 3809 | | 3812 | | 3814 | | 3822 | | 3838 | | 3849 | | 3852 | | 3865 | |  |
| 2702009 | COITÉ DO NÓIA | | INNER CITY | | 5901 | | 5835 | | 5773 | | 5712 | | 5655 | | 5612 | | 5571 | | 5531 | | 5466 | | 5423 | |  |
| 2702108 | COLÔNIA LEOPOLDINA | | INNER CITY | | 10100 | | 10214 | | 10315 | | 10420 | | 10508 | | 10607 | | 10699 | | 10781 | | 10831 | | 10886 | |  |
| 2702207 | COQUEIRO SECO | | METRO REGION | | 2811 | | 2834 | | 2852 | | 2864 | | 2875 | | 2889 | | 2892 | | 2899 | | 2913 | | 2922 | |  |
| 2702306 | CORURIPE | | INNER CITY | | 25653 | | 26005 | | 26334 | | 26647 | | 26926 | | 27173 | | 27310 | | 27519 | | 27747 | | 27926 | |  |
| 2702355 | CRAÍBAS | | INNER CITY | | 11332 | | 11432 | | 11522 | | 11603 | | 11680 | | 11734 | | 11797 | | 11854 | | 11991 | | 12046 | |  |
| 2702405 | DELMIRO GOUVEIA | | INNER CITY | | 23283 | | 23523 | | 23754 | | 23962 | | 24157 | | 24323 | | 24496 | | 24654 | | 24788 | | 25071 | |  |
| 2702504 | DOIS RIACHOS | | INNER CITY | | 5639 | | 5653 | | 5663 | | 5672 | | 5686 | | 5683 | | 5651 | | 5643 | | 5657 | | 5651 | |  |
| 2702553 | ESTRELA DE ALAGOAS | | INNER CITY | | 8540 | | 8605 | | 8666 | | 8727 | | 8780 | | 8838 | | 8898 | | 8950 | | 8997 | | 9066 | |  |
| 2702603 | FEIRA GRANDE | | INNER CITY | | 10993 | | 11006 | | 11021 | | 11027 | | 11031 | | 11014 | | 10985 | | 11073 | | 11074 | | 11056 | |  |
| 2702702 | FELIZ DESERTO | | INNER CITY | | 2154 | | 2176 | | 2190 | | 2210 | | 2224 | | 2234 | | 2304 | | 2312 | | 2322 | | 2328 | |  |
| 2702801 | FLEXEIRAS | | INNER CITY | | 6497 | | 6499 | | 6505 | | 6507 | | 6514 | | 6507 | | 6507 | | 6511 | | 6500 | | 6494 | |  |
| 2702900 | GIRAU DO PONCIANO | | INNER CITY | | 17824 | | 18153 | | 18463 | | 18750 | | 19020 | | 19243 | | 19639 | | 19878 | | 20079 | | 20290 | |  |
| 2703007 | IBATEGUARA | | INNER CITY | | 7840 | | 7860 | | 7874 | | 7896 | | 7909 | | 7918 | | 7923 | | 7932 | | 7959 | | 7975 | |  |
| 2703106 | IGACI | | INNER CITY | | 12898 | | 12880 | | 12861 | | 12839 | | 12817 | | 12790 | | 12762 | | 12734 | | 12708 | | 12674 | |  |
| 2703205 | IGREJA NOVA | | INNER CITY | | 11930 | | 12029 | | 12124 | | 12209 | | 12288 | | 12377 | | 12462 | | 12458 | | 12518 | | 12597 | |  |
| 2703304 | INHAPI | | INNER CITY | | 9126 | | 9142 | | 9156 | | 9175 | | 9191 | | 9230 | | 9179 | | 9204 | | 9208 | | 9257 | |  |
| 2703403 | JACARÉ DOS HOMENS | | INNER CITY | | 2934 | | 2897 | | 2862 | | 2829 | | 2802 | | 2770 | | 2746 | | 2728 | | 2698 | | 2669 | |  |
| 2703502 | JACUÍPE | | INNER CITY | | 3804 | | 3786 | | 3769 | | 3753 | | 3728 | | 3709 | | 3682 | | 3650 | | 3646 | | 3626 | |  |
| 2703601 | JAPARATINGA | | INNER CITY | | 3920 | | 3957 | | 3991 | | 4013 | | 4042 | | 4065 | | 4090 | | 4112 | | 4116 | | 4123 | |  |
| 2703700 | JARAMATAIA | | INNER CITY | | 2952 | | 2943 | | 2934 | | 2930 | | 2931 | | 2927 | | 2922 | | 2917 | | 2921 | | 2917 | |  |
| 2703759 | JEQUIÁ DA PRAIA | | INNER CITY | | 6594 | | 6541 | | 6487 | | 6442 | | 6391 | | 6354 | | 6311 | | 6144 | | 6048 | | 5973 | |  |
| 2703809 | JOAQUIM GOMES | | INNER CITY | | 11729 | | 11814 | | 11899 | | 11974 | | 12038 | | 12087 | | 12157 | | 12219 | | 12237 | | 12277 | |  |
| 2703908 | JUNDIÁ | | INNER CITY | | 2322 | | 2299 | | 2270 | | 2244 | | 2218 | | 2188 | | 2163 | | 2139 | | 2124 | | 2123 | |  |
| 2704005 | JUNQUEIRO | | INNER CITY | | 12339 | | 12323 | | 12308 | | 12288 | | 12268 | | 12261 | | 12464 | | 12460 | | 12434 | | 12425 | |  |
| 2704104 | LAGOA DA CANOA | | INNER CITY | | 9673 | | 9593 | | 9521 | | 9448 | | 9386 | | 9318 | | 9255 | | 9199 | | 9123 | | 9067 | |  |
| 2704203 | LIMOEIRO DE ANADIA | | INNER CITY | | 13447 | | 13610 | | 13757 | | 13899 | | 14026 | | 14157 | | 14080 | | 14184 | | 14288 | | 14391 | |  |
| 2704302 | MACEIÓ (Capital) | | METRO REGION | | 436268 | | 442365 | | 448032 | | 453249 | | 458024 | | 462471 | | 466681 | | 470419 | | 473643 | | 476852 | |  |
| 2704401 | MAJOR ISIDORO | | INNER CITY | | 9471 | | 9540 | | 9592 | | 9645 | | 9694 | | 9729 | | 9766 | | 9817 | | 9857 | | 9895 | |  |
| 2704906 | MAR VERMELHO | | INNER CITY | | 1988 | | 1967 | | 1941 | | 1920 | | 1900 | | 1883 | | 1868 | | 1851 | | 1827 | | 1794 | |  |
| 2704500 | MARAGOGI | | INNER CITY | | 13928 | | 14263 | | 14589 | | 14885 | | 15163 | | 15419 | | 15657 | | 15877 | | 16083 | | 16275 | |  |
| 2704609 | MARAVILHA | | INNER CITY | | 6002 | | 5846 | | 5694 | | 5557 | | 5428 | | 5297 | | 5279 | | 5170 | | 5052 | | 4948 | |  |
| 2704708 | MARECHAL DEODORO | | METRO REGION | | 21947 | | 22453 | | 22927 | | 23369 | | 23775 | | 24120 | | 24446 | | 24742 | | 25065 | | 25343 | |  |
| 2704807 | MARIBONDO | | INNER CITY | | 7105 | | 7034 | | 6971 | | 6906 | | 6844 | | 6783 | | 6730 | | 6677 | | 6636 | | 6607 | |  |
| 2705002 | MATA GRANDE | | INNER CITY | | 12863 | | 12861 | | 12858 | | 12862 | | 12860 | | 12876 | | 12760 | | 12758 | | 12939 | | 12919 | |  |
| 2705101 | MATRIZ DE CAMARAGIBE | | INNER CITY | | 12241 | | 12211 | | 12189 | | 12166 | | 12151 | | 12116 | | 12109 | | 12326 | | 12298 | | 12300 | |  |
| 2705200 | MESSIAS | | METRO REGION | | 7433 | | 7606 | | 7770 | | 7921 | | 8065 | | 8202 | | 8339 | | 8464 | | 8567 | | 8674 | |  |
| 2705309 | MINADOR DO NEGRÃO | | INNER CITY | | 2762 | | 2753 | | 2745 | | 2739 | | 2732 | | 2720 | | 2711 | | 2698 | | 2691 | | 2680 | |  |
| 2705408 | MONTEIRÓPOLIS | | INNER CITY | | 3495 | | 3501 | | 3504 | | 3508 | | 3513 | | 3509 | | 3533 | | 3535 | | 3539 | | 3552 | |  |
| 2705507 | MURICI | | METRO REGION | | 13521 | | 13616 | | 13707 | | 13793 | | 13867 | | 13942 | | 14007 | | 14064 | | 14066 | | 14122 | |  |
| 2705606 | NOVO LINO | | INNER CITY | | 6113 | | 6192 | | 6264 | | 6326 | | 6393 | | 6444 | | 6498 | | 6340 | | 6393 | | 6412 | |  |
| 2705705 | OLHO D'ÁGUA DAS FLORES | | INNER CITY | | 9986 | | 10040 | | 10085 | | 10132 | | 10169 | | 10202 | | 10240 | | 10386 | | 10416 | | 10450 | |  |
| 2705804 | OLHO D'ÁGUA DO CASADO | | INNER CITY | | 4192 | | 4264 | | 4337 | | 4403 | | 4465 | | 4514 | | 4565 | | 4609 | | 4662 | | 4705 | |  |
| 2705903 | OLHO D'ÁGUA GRANDE | | INNER CITY | | 2528 | | 2540 | | 2544 | | 2551 | | 2551 | | 2564 | | 2539 | | 2528 | | 2544 | | 2545 | |  |
| 2705903 | OLIVENÇA | | INNER CITY | | 5722 | | 5748 | | 5774 | | 5798 | | 5818 | | 5834 | | 5864 | | 5885 | | 5891 | | 5906 | |  |
| 2706000 | OURO BRANCO | | INNER CITY | | 5552 | | 5592 | | 5628 | | 5657 | | 5685 | | 5705 | | 5672 | | 5698 | | 5721 | | 5738 | |  |
| 2706109 | PALESTINA | | INNER CITY | | 2468 | | 2499 | | 2529 | | 2556 | | 2579 | | 2606 | | 2594 | | 2343 | | 2376 | | 2378 | |  |
| 2706109 | PALMEIRA DOS ÍNDIOS | | INNER CITY | | 34781 | | 34885 | | 34983 | | 35068 | | 35144 | | 35192 | | 35231 | | 35257 | | 35287 | | 35288 | |  |
| 2706307 | PÃO DE AÇÚCAR | | INNER CITY | | 12413 | | 12378 | | 12345 | | 12321 | | 12299 | | 12258 | | 12232 | | 12451 | | 12435 | | 12413 | |  |
| 2706406 | PARICONHA | | INNER CITY | | 5303 | | 5321 | | 5334 | | 5343 | | 5355 | | 5346 | | 5352 | | 5361 | | 5363 | | 5322 | |  |
| 2706422 | PARIPUEIRA | | METRO REGION | | 5244 | | 5408 | | 5568 | | 5720 | | 5862 | | 5982 | | 6110 | | 6228 | | 6345 | | 6453 | |  |
| 2706448 | PASSO DE CAMARAGIBE | | INNER CITY | | 7806 | | 7819 | | 7830 | | 7847 | | 7853 | | 7859 | | 7869 | | 7881 | | 7889 | | 7896 | |  |
| 2706505 | PAULO JACINTO | | INNER CITY | | 3800 | | 3796 | | 3796 | | 3792 | | 3792 | | 3792 | | 3784 | | 3773 | | 3767 | | 3757 | |  |
| 2706604 | PENEDO | | INNER CITY | | 30115 | | 30273 | | 30412 | | 30538 | | 30649 | | 30780 | | 30873 | | 31067 | | 31142 | | 31216 | |  |
| 2706703 | PIAÇABUÇU | | INNER CITY | | 8860 | | 8900 | | 8936 | | 8973 | | 9008 | | 9046 | | 9071 | | 9098 | | 9140 | | 9176 | |  |
| 2706802 | PILAR | | METRO REGION | | 16585 | | 16673 | | 16759 | | 16836 | | 16912 | | 16964 | | 17001 | | 17030 | | 17102 | | 17150 | |  |
| 2706901 | PINDOBA | | INNER CITY | | 1510 | | 1501 | | 1491 | | 1485 | | 1473 | | 1479 | | 1477 | | 1472 | | 1461 | | 1456 | |  |
| 2706901 | PIRANHAS | | INNER CITY | | 11280 | | 11421 | | 11561 | | 11684 | | 11804 | | 11912 | | 12016 | | 12111 | | 12195 | | 12274 | |  |
| 2707107 | POÇO DAS TRINCHEIRAS | | INNER CITY | | 7200 | | 7233 | | 7257 | | 7279 | | 7302 | | 7326 | | 7302 | | 7324 | | 7334 | | 7348 | |  |
| 2707206 | PORTO CALVO | | INNER CITY | | 13039 | | 13111 | | 13180 | | 13235 | | 13285 | | 13355 | | 13378 | | 13393 | | 13447 | | 13477 | |  |
| 2707305 | PORTO DE PEDRAS | | INNER CITY | | 4821 | | 4723 | | 4626 | | 4540 | | 4453 | | 4382 | | 4312 | | 4239 | | 4177 | | 4118 | |  |
| 2707404 | PORTO REAL DO COLÉGIO | | INNER CITY | | 9830 | | 9881 | | 9923 | | 9966 | | 10003 | | 10052 | | 9965 | | 9996 | | 10023 | | 10047 | |  |
| 2707503 | QUEBRANGULO | | INNER CITY | | 6123 | | 6068 | | 6019 | | 5975 | | 5925 | | 5886 | | 5845 | | 5806 | | 5765 | | 5729 | |  |
| 2707602 | RIO LARGO | | METRO REGION | | 33976 | | 34234 | | 34466 | | 34675 | | 34869 | | 35040 | | 35031 | | 35180 | | 36805 | | 36942 | |  |
| 2707701 | ROTEIRO | | INNER CITY | | 3551 | | 3543 | | 3535 | | 3531 | | 3524 | | 3501 | | 3490 | | 3472 | | 3477 | | 3467 | |  |
| 2707800 | SANTA LUZIA DO NORTE | | METRO REGION | | 3440 | | 3463 | | 3490 | | 3508 | | 3523 | | 3546 | | 3556 | | 3571 | | 3587 | | 3598 | |  |
| 2707909 | SANTANA DO IPANEMA | | INNER CITY | | 22246 | | 22413 | | 22565 | | 22715 | | 22858 | | 22996 | | 23170 | | 23326 | | 23417 | | 23545 | |  |
| 2708006 | SANTANA DO MUNDAÚ | | INNER CITY | | 5923 | | 5873 | | 5827 | | 5781 | | 5739 | | 5689 | | 5642 | | 5604 | | 5565 | | 5528 | |  |
| 2708105 | SÃO BRÁS | | INNER CITY | | 3474 | | 3476 | | 3478 | | 3483 | | 3480 | | 3486 | | 3484 | | 3481 | | 3485 | | 3488 | |  |
| 2708204 | SÃO JOSÉ DA LAJE | | INNER CITY | | 11429 | | 11502 | | 11564 | | 11622 | | 11676 | | 11735 | | 11801 | | 11855 | | 11892 | | 11939 | |  |
| 2708303 | SÃO JOSÉ DA TAPERA | | INNER CITY | | 14959 | | 15110 | | 15249 | | 15373 | | 15489 | | 15620 | | 15741 | | 15859 | | 15938 | | 16034 | |  |
| 2708402 | SÃO LUÍS DO QUITUNDE | | INNER CITY | | 16561 | | 16700 | | 16832 | | 16955 | | 17066 | | 17167 | | 17263 | | 17353 | | 17443 | | 17526 | |  |
| 2708501 | SÃO MIGUEL DOS CAMPOS | | INNER CITY | | 25851 | | 26393 | | 26906 | | 27381 | | 27824 | | 28238 | | 28640 | | 29016 | | 29386 | | 29724 | |  |
| 2708600 | SÃO MIGUEL DOS MILAGRES | | INNER CITY | | 3509 | | 3570 | | 3629 | | 3674 | | 3716 | | 3766 | | 3799 | | 3838 | | 3863 | | 3890 | |  |
| 2708709 | SÃO SEBASTIÃO | | INNER CITY | | 16084 | | 16217 | | 16342 | | 16462 | | 16569 | | 16643 | | 16716 | | 16778 | | 16849 | | 16906 | |  |
| 2708808 | SATUBA | | METRO REGION | | 6976 | | 7106 | | 7226 | | 7329 | | 7426 | | 7540 | | 7614 | | 7695 | | 6463 | | 6521 | |  |
| 2708907 | SENADOR RUI PALMEIRA | | INNER CITY | | 6520 | | 6559 | | 6599 | | 6639 | | 6673 | | 6705 | | 6740 | | 6763 | | 6778 | | 6797 | |  |
| 2708956 | TANQUE D'ARCA | | INNER CITY | | 3239 | | 3219 | | 3192 | | 3170 | | 3144 | | 3132 | | 3173 | | 3157 | | 3134 | | 3115 | |  |
| 2709004 | TAQUARANA | | INNER CITY | | 9361 | | 9441 | | 9513 | | 9581 | | 9646 | | 9706 | | 9546 | | 9600 | | 9622 | | 9652 | |  |
| 2709103 | TEOTÔNIO VILELA | | INNER CITY | | 20438 | | 20618 | | 20784 | | 20937 | | 21080 | | 21195 | | 21325 | | 21423 | | 21514 | | 21604 | |  |
| 2709152 | TRAIPU | | INNER CITY | | 13068 | | 13192 | | 13309 | | 13423 | | 13530 | | 13610 | | 13853 | | 13961 | | 14047 | | 14132 | |  |
| 2709202 | UNIÃO DOS PALMARES | | INNER CITY | | 31010 | | 31168 | | 31308 | | 31430 | | 31537 | | 31679 | | 31800 | | 31918 | | 31975 | | 32056 | |  |
| 2709301 | VIÇOSA | | INNER CITY | | 13134 | | 13091 | | 13052 | | 13013 | | 12975 | | 12937 | | 13008 | | 12965 | | 12938 | | 12914 | |  |
|  | |  | |  | | **FEMALE POPULATION** | | | | | | | | | | | | | | | | | | | |
|  | |  | |  | | **2006** | | **2007** | | **2008** | | **2009** | | **2010** | | **2011** | | **2012** | | **2013** | | **2014** | | **2015** | |
| 2700102 | | ÁGUA BRANCA | | INNER CITY | | 9846 | | 9878 | | 9916 | | 9950 | | 9985 | | 10023 | | 10165 | | 10193 | | 10218 | | 10129 | |
| 2700201 | | ANADIA | | INNER CITY | | 9059 | | 9075 | | 9083 | | 9099 | | 9112 | | 9092 | | 9094 | | 9098 | | 9113 | | 9067 | |
| 2700300 | | ARAPIRACA | | INNER CITY | | 109222 | | 110751 | | 112216 | | 113609 | | 114938 | | 116200 | | 117380 | | 118397 | | 119411 | | 120438 | |
| 2700409 | | ATALAIA | | INNER CITY | | 21844 | | 22078 | | 22296 | | 22508 | | 22706 | | 22912 | | 23098 | | 23287 | | 23459 | | 23635 | |
| 2700508 | | BARRA DE SANTO ANTÔNIO | | METRO REGION | | 6746 | | 6904 | | 7056 | | 7199 | | 7331 | | 7468 | | 7588 | | 7708 | | 7823 | | 7937 | |
| 2700607 | | BARRA DE SÃO MIGUEL | | METRO REGION | | 3678 | | 3735 | | 3789 | | 3842 | | 3890 | | 3940 | | 3975 | | 4007 | | 4040 | | 4066 | |
| 2700706 | | BATALHA | | METRO REGION | | 8470 | | 8588 | | 8697 | | 8806 | | 8912 | | 9009 | | 9132 | | 9215 | | 9297 | | 9380 | |
| 2700805 | | BELÉM | | INNER CITY | | 2636 | | 2568 | | 2506 | | 2447 | | 2389 | | 2342 | | 2448 | | 2404 | | 2363 | | 2326 | |
| 2700904 | | BELO MONTE | | INNER CITY | | 3561 | | 3567 | | 3574 | | 3581 | | 3590 | | 3581 | | 3293 | | 3307 | | 3290 | | 3288 | |
| 2701001 | | BOCA DA MATA | | INNER CITY | | 12953 | | 13055 | | 13159 | | 13256 | | 13348 | | 13448 | | 13546 | | 13641 | | 13716 | | 13853 | |
| 2701100 | | BRANQUINHA | | INNER CITY | | 5413 | | 5400 | | 5379 | | 5359 | | 5340 | | 5336 | | 5323 | | 5313 | | 5296 | | 5287 | |
| 2701209 | | CACIMBINHAS | | INNER CITY | | 5193 | | 5217 | | 5246 | | 5270 | | 5298 | | 5315 | | 5343 | | 5369 | | 5382 | | 5394 | |
| 2701308 | | CAJUEIRO | | INNER CITY | | 10358 | | 10430 | | 10497 | | 10565 | | 10616 | | 10668 | | 10775 | | 10838 | | 10707 | | 10762 | |
| 2701357 | | CAMPESTRE | | INNER CITY | | 3328 | | 3350 | | 3363 | | 3383 | | 3403 | | 3425 | | 3442 | | 3467 | | 3479 | | 3499 | |
| 2701407 | | CAMPO ALEGRE | | INNER CITY | | 24674 | | 25166 | | 25631 | | 26058 | | 26459 | | 26840 | | 27204 | | 27719 | | 28043 | | 28361 | |
| 2701506 | | CAMPO GRANDE | | INNER CITY | | 4640 | | 4662 | | 4682 | | 4701 | | 4714 | | 4735 | | 4814 | | 4831 | | 4840 | | 4839 | |
| 2701605 | | CANAPI | | INNER CITY | | 8830 | | 8823 | | 8824 | | 8818 | | 8815 | | 8811 | | 8792 | | 8776 | | 8811 | | 8796 | |
| 2701704 | | CAPELA | | INNER CITY | | 9084 | | 9026 | | 8965 | | 8915 | | 8871 | | 8843 | | 8757 | | 8717 | | 8901 | | 8874 | |
| 2701803 | | CARNEIROS | | INNER CITY | | 4012 | | 4108 | | 4195 | | 4282 | | 4357 | | 4420 | | 4485 | | 4460 | | 4521 | | 4586 | |
| 2701902 | | CHÃ PRETA | | INNER CITY | | 3576 | | 3581 | | 3582 | | 3584 | | 3587 | | 3583 | | 3572 | | 3564 | | 3564 | | 3555 | |
| 2702009 | | COITÉ DO NÓIA | | INNER CITY | | 5757 | | 5730 | | 5703 | | 5682 | | 5661 | | 5631 | | 5604 | | 5579 | | 5576 | | 5569 | |
| 2702108 | | COLÔNIA LEOPOLDINA | | INNER CITY | | 9679 | | 9825 | | 9970 | | 10097 | | 10225 | | 10329 | | 10428 | | 10526 | | 10643 | | 10743 | |
| 2702207 | | COQUEIRO SECO | | INNER CITY | | 2756 | | 2776 | | 2798 | | 2824 | | 2848 | | 2867 | | 2895 | | 2918 | | 2930 | | 2945 | |
| 2702306 | | CORURIPE | | METRO REGION | | 25481 | | 25909 | | 26316 | | 26695 | | 27064 | | 27425 | | 27799 | | 28129 | | 28398 | | 28696 | |
| 2702355 | | CRAÍBAS | | INNER CITY | | 11393 | | 11490 | | 11587 | | 11682 | | 11769 | | 11869 | | 11952 | | 12031 | | 12171 | | 12238 | |
| 2702405 | | DELMIRO GOUVEIA | | INNER CITY | | 24557 | | 24855 | | 25133 | | 25403 | | 25655 | | 25909 | | 26131 | | 26345 | | 26553 | | 26917 | |
| 2702504 | | DOIS RIACHOS | | INNER CITY | | 5661 | | 5638 | | 5620 | | 5603 | | 5582 | | 5578 | | 5589 | | 5591 | | 5569 | | 5570 | |
| 2702553 | | ESTRELA DE ALAGOAS | | INNER CITY | | 8902 | | 8953 | | 9002 | | 9044 | | 9087 | | 9119 | | 9145 | | 9173 | | 9196 | | 9237 | |
| 2702603 | | FEIRA GRANDE | | INNER CITY | | 10946 | | 10972 | | 10994 | | 11023 | | 11051 | | 11098 | | 11144 | | 11304 | | 11327 | | 11371 | |
| 2702702 | | FELIZ DESERTO | | INNER CITY | | 2153 | | 2184 | | 2219 | | 2246 | | 2276 | | 2307 | | 2337 | | 2366 | | 2393 | | 2415 | |
| 2702801 | | FLEXEIRAS | | INNER CITY | | 6107 | | 6149 | | 6185 | | 6222 | | 6251 | | 6292 | | 6325 | | 6351 | | 6387 | | 6421 | |
| 2702900 | | GIRAU DO PONCIANO | | INNER CITY | | 17587 | | 17939 | | 18272 | | 18590 | | 18886 | | 19194 | | 19546 | | 19779 | | 20016 | | 20227 | |
| 2703007 | | IBATEGUARA | | INNER CITY | | 7730 | | 7743 | | 7760 | | 7767 | | 7781 | | 7797 | | 7816 | | 7830 | | 7824 | | 7826 | |
| 2703106 | | IGACI | | INNER CITY | | 13249 | | 13251 | | 13254 | | 13262 | | 13270 | | 13284 | | 13300 | | 13317 | | 13348 | | 13362 | |
| 2703205 | | IGREJA NOVA | | INNER CITY | | 11471 | | 11569 | | 11660 | | 11750 | | 11835 | | 11900 | | 11960 | | 11870 | | 11936 | | 11972 | |
| 2703304 | | INHAPI | | INNER CITY | | 9309 | | 9321 | | 9333 | | 9339 | | 9346 | | 9329 | | 9318 | | 9312 | | 9320 | | 9363 | |
| 2703403 | | JACARÉ DOS HOMENS | | INNER CITY | | 2914 | | 2885 | | 2857 | | 2832 | | 2804 | | 2785 | | 2811 | | 2783 | | 2770 | | 2760 | |
| 2703502 | | JACUÍPE | | INNER CITY | | 3532 | | 3526 | | 3520 | | 3514 | | 3519 | | 3519 | | 3528 | | 3543 | | 3531 | | 3535 | |
| 2703601 | | JAPARATINGA | | INNER CITY | | 3774 | | 3829 | | 3882 | | 3942 | | 3989 | | 4038 | | 4080 | | 4122 | | 4180 | | 4226 | |
| 2703700 | | JARAMATAIA | | INNER CITY | | 2868 | | 2859 | | 2852 | | 2841 | | 2825 | | 2815 | | 2808 | | 2801 | | 2782 | | 2775 | |
| 2703759 | | JEQUIÁ DA PRAIA | | INNER CITY | | 6160 | | 6132 | | 6110 | | 6083 | | 6067 | | 6041 | | 6025 | | 5825 | | 5865 | | 5893 | |
| 2703809 | | JOAQUIM GOMES | | INNER CITY | | 10931 | | 11043 | | 11143 | | 11243 | | 11342 | | 11446 | | 11521 | | 11594 | | 11694 | | 11783 | |
| 2703908 | | JUNDIÁ | | INNER CITY | | 2158 | | 2146 | | 2142 | | 2137 | | 2134 | | 2137 | | 2136 | | 2136 | | 2130 | | 2149 | |
| 2704005 | | JUNQUEIRO | | INNER CITY | | 12331 | | 12352 | | 12371 | | 12394 | | 12418 | | 12428 | | 12603 | | 12613 | | 12643 | | 12659 | |
| 2704104 | | LAGOA DA CANOA | | INNER CITY | | 9784 | | 9712 | | 9641 | | 9579 | | 9515 | | 9465 | | 9416 | | 9367 | | 9308 | | 9276 | |
| 2704203 | | LIMOEIRO DE ANADIA | | INNER CITY | | 13400 | | 13540 | | 13678 | | 13805 | | 13929 | | 14034 | | 13958 | | 14060 | | 14148 | | 14230 | |
| 2704302 | | MACEIÓ (Capital) | | INNER CITY | | 479210 | | 486911 | | 494276 | | 501310 | | 508005 | | 514327 | | 520518 | | 526314 | | 531548 | | 536791 | |
| 2704401 | | MAJOR ISIDORO | | METRO REGION | | 9597 | | 9665 | | 9743 | | 9812 | | 9877 | | 9949 | | 10013 | | 10057 | | 10104 | | 10146 | |
| 2704906 | | MAR VERMELHO | | INNER CITY | | 1933 | | 1916 | | 1906 | | 1894 | | 1882 | | 1869 | | 1857 | | 1847 | | 1847 | | 1824 | |
| 2704500 | | MARAGOGI | | INNER CITY | | 13316 | | 13672 | | 13999 | | 14316 | | 14612 | | 14895 | | 15164 | | 15422 | | 15662 | | 15892 | |
| 2704609 | | MARAVILHA | | INNER CITY | | 5817 | | 5654 | | 5505 | | 5359 | | 5223 | | 5105 | | 5107 | | 4998 | | 4908 | | 4825 | |
| 2704708 | | MARECHAL DEODORO | | INNER CITY | | 21959 | | 22466 | | 22949 | | 23407 | | 23843 | | 24289 | | 24707 | | 25111 | | 25442 | | 25784 | |
| 2704807 | | MARIBONDO | | METRO REGION | | 7496 | | 7431 | | 7367 | | 7311 | | 7261 | | 7216 | | 7170 | | 7130 | | 7084 | | 7055 | |
| 2705002 | | MATA GRANDE | | INNER CITY | | 12757 | | 12747 | | 12740 | | 12726 | | 12719 | | 12694 | | 12596 | | 12591 | | 12717 | | 12666 | |
| 2705101 | | MATRIZ DE CAMARAGIBE | | INNER CITY | | 12400 | | 12428 | | 12448 | | 12470 | | 12483 | | 12516 | | 12522 | | 12679 | | 12705 | | 12704 | |
| 2705200 | | MESSIAS | | INNER CITY | | 7457 | | 7653 | | 7838 | | 8014 | | 8177 | | 8328 | | 8515 | | 8646 | | 8779 | | 8903 | |
| 2705309 | | MINADOR DO NEGRÃO | | METRO REGION | | 2741 | | 2739 | | 2737 | | 2733 | | 2731 | | 2735 | | 2736 | | 2741 | | 2741 | | 2746 | |
| 2705408 | | MONTEIRÓPOLIS | | INNER CITY | | 3623 | | 3634 | | 3648 | | 3659 | | 3669 | | 3687 | | 3674 | | 3684 | | 3689 | | 3691 | |
| 2705507 | | MURICI | | INNER CITY | | 13339 | | 13463 | | 13579 | | 13688 | | 13796 | | 13892 | | 14000 | | 14094 | | 14128 | | 14214 | |
| 2705606 | | NOVO LINO | | METRO REGION | | 5773 | | 5859 | | 5942 | | 6027 | | 6097 | | 6175 | | 6242 | | 6139 | | 6187 | | 6228 | |
| 2705705 | | OLHO D'ÁGUA DAS FLORES | | INNER CITY | | 10707 | | 10762 | | 10819 | | 10869 | | 10922 | | 10974 | | 10966 | | 11113 | | 11156 | | 11186 | |
| 2705804 | | OLHO D'ÁGUA DO CASADO | | INNER CITY | | 4070 | | 4143 | | 4207 | | 4270 | | 4329 | | 4393 | | 4449 | | 4505 | | 4547 | | 4592 | |
| 2705903 | | OLHO D'ÁGUA GRANDE | | INNER CITY | | 2552 | | 2555 | | 2565 | | 2571 | | 2583 | | 2582 | | 2610 | | 2631 | | 2625 | | 2634 | |
| 2705903 | | OLIVENÇA | | INNER CITY | | 5445 | | 5494 | | 5538 | | 5581 | | 5623 | | 5665 | | 5690 | | 5709 | | 5749 | | 5775 | |
| 2706000 | | OURO BRANCO | | INNER CITY | | 5420 | | 5470 | | 5519 | | 5569 | | 5616 | | 5666 | | 5677 | | 5711 | | 5759 | | 5796 | |
| 2706109 | | PALESTINA | | INNER CITY | | 2602 | | 2632 | | 2660 | | 2687 | | 2715 | | 2736 | | 2793 | | 2591 | | 2594 | | 2625 | |
| 2706109 | | PALMEIRA DOS ÍNDIOS | | INNER CITY | | 37013 | | 37205 | | 37387 | | 37565 | | 37735 | | 37918 | | 38097 | | 38275 | | 38430 | | 38579 | |
| 2706307 | | PÃO DE AÇÚCAR | | INNER CITY | | 12549 | | 12502 | | 12457 | | 12408 | | 12362 | | 12339 | | 12305 | | 12524 | | 12488 | | 12458 | |
| 2706406 | | PARICONHA | | INNER CITY | | 5253 | | 5255 | | 5261 | | 5270 | | 5275 | | 5300 | | 5309 | | 5313 | | 5321 | | 5279 | |
| 2706422 | | PARIPUEIRA | | INNER CITY | | 5309 | | 5472 | | 5621 | | 5760 | | 5890 | | 6025 | | 6138 | | 6246 | | 6343 | | 6432 | |
| 2706448 | | PASSO DE CAMARAGIBE | | METRO REGION | | 7348 | | 7372 | | 7396 | | 7412 | | 7437 | | 7460 | | 7477 | | 7491 | | 7503 | | 7524 | |
| 2706505 | | PAULO JACINTO | | INNER CITY | | 3901 | | 3902 | | 3900 | | 3901 | | 3899 | | 3897 | | 3903 | | 3912 | | 3915 | | 3919 | |
| 2706604 | | PENEDO | | INNER CITY | | 31033 | | 31253 | | 31471 | | 31680 | | 31883 | | 32047 | | 32231 | | 32528 | | 32690 | | 32854 | |
| 2706703 | | PIAÇABUÇU | | INNER CITY | | 8752 | | 8768 | | 8785 | | 8797 | | 8809 | | 8815 | | 8831 | | 8843 | | 8834 | | 8835 | |
| 2706802 | | PILAR | | INNER CITY | | 17061 | | 17204 | | 17337 | | 17465 | | 17581 | | 17709 | | 17842 | | 17973 | | 18047 | | 18140 | |
| 2706901 | | PINDOBA | | METRO REGION | | 1470 | | 1476 | | 1483 | | 1486 | | 1495 | | 1486 | | 1486 | | 1489 | | 1497 | | 1501 | |
| 2706901 | | PIRANHAS | | INNER CITY | | 11443 | | 11614 | | 11769 | | 11923 | | 12063 | | 12199 | | 12324 | | 12445 | | 12561 | | 12674 | |
| 2707107 | | POÇO DAS TRINCHEIRAS | | INNER CITY | | 6894 | | 6936 | | 6982 | | 7026 | | 7065 | | 7099 | | 7048 | | 7077 | | 7116 | | 7143 | |
| 2707206 | | PORTO CALVO | | INNER CITY | | 12886 | | 13005 | | 13116 | | 13231 | | 13340 | | 13419 | | 13537 | | 13654 | | 13722 | | 13809 | |
| 2707305 | | PORTO DE PEDRAS | | INNER CITY | | 4521 | | 4452 | | 4391 | | 4329 | | 4277 | | 4218 | | 4165 | | 4123 | | 4075 | | 4033 | |
| 2707404 | | PORTO REAL DO COLÉGIO | | INNER CITY | | 9788 | | 9848 | | 9910 | | 9966 | | 10021 | | 10059 | | 10025 | | 10070 | | 10112 | | 10158 | |
| 2707503 | | QUEBRANGULO | | INNER CITY | | 6082 | | 6051 | | 6019 | | 5987 | | 5965 | | 5937 | | 5915 | | 5894 | | 5877 | | 5861 | |
| 2707602 | | RIO LARGO | | INNER CITY | | 34877 | | 35184 | | 35486 | | 35779 | | 36055 | | 36325 | | 36414 | | 36654 | | 38451 | | 38696 | |
| 2707701 | | ROTEIRO | | METRO REGION | | 3437 | | 3419 | | 3403 | | 3384 | | 3369 | | 3372 | | 3364 | | 3364 | | 3344 | | 3336 | |
| 2707800 | | SANTA LUZIA DO NORTE | | INNER CITY | | 3498 | | 3529 | | 3554 | | 3584 | | 3614 | | 3633 | | 3663 | | 3686 | | 3707 | | 3727 | |
| 2707909 | | SANTANA DO IPANEMA | | METRO REGION | | 22932 | | 23136 | | 23333 | | 23512 | | 23677 | | 23828 | | 23926 | | 24026 | | 24169 | | 24268 | |
| 2708006 | | SANTANA DO MUNDAÚ | | INNER CITY | | 5791 | | 5742 | | 5695 | | 5653 | | 5613 | | 5586 | | 5560 | | 5530 | | 5503 | | 5484 | |
| 2708105 | | SÃO BRÁS | | INNER CITY | | 3404 | | 3424 | | 3442 | | 3457 | | 3478 | | 3489 | | 3507 | | 3525 | | 3536 | | 3547 | |
| 2708204 | | SÃO JOSÉ DA LAJE | | INNER CITY | | 11482 | | 11568 | | 11657 | | 11740 | | 11819 | | 11884 | | 11936 | | 11992 | | 12056 | | 12109 | |
| 2708303 | | SÃO JOSÉ DA TAPERA | | INNER CITY | | 15032 | | 15201 | | 15364 | | 15523 | | 15673 | | 15791 | | 15905 | | 16008 | | 16134 | | 16234 | |
| 2708402 | | SÃO LUÍS DO QUITUNDE | | INNER CITY | | 15893 | | 16058 | | 16213 | | 16360 | | 16502 | | 16638 | | 16766 | | 16886 | | 16989 | | 17091 | |
| 2708501 | | SÃO MIGUEL DOS CAMPOS | | INNER CITY | | 26433 | | 27048 | | 27628 | | 28181 | | 28700 | | 29189 | | 29637 | | 30061 | | 30439 | | 30808 | |
| 2708600 | | SÃO MIGUEL DOS MILAGRES | | INNER CITY | | 3428 | | 3498 | | 3564 | | 3636 | | 3703 | | 3756 | | 3819 | | 3871 | | 3930 | | 3983 | |
| 2708709 | | SÃO SEBASTIÃO | | INNER CITY | | 15949 | | 16122 | | 16285 | | 16436 | | 16583 | | 16747 | | 16899 | | 17048 | | 17170 | | 17302 | |
| 2708808 | | SATUBA | | INNER CITY | | 7130 | | 7278 | | 7420 | | 7564 | | 7698 | | 7801 | | 7931 | | 8042 | | 6775 | | 6857 | |
| 2708907 | | SENADOR RUI PALMEIRA | | METRO REGION | | 6575 | | 6650 | | 6718 | | 6779 | | 6840 | | 6897 | | 6946 | | 7002 | | 7056 | | 7109 | |
| 2708956 | | TANQUE D'ARCA | | INNER CITY | | 3248 | | 3228 | | 3217 | | 3203 | | 3196 | | 3177 | | 3232 | | 3217 | | 3211 | | 3203 | |
| 2709004 | | TAQUARANA | | INNER CITY | | 9582 | | 9708 | | 9831 | | 9946 | | 10053 | | 10154 | | 10040 | | 10125 | | 10229 | | 10324 | |
| 2709103 | | TEOTÔNIO VILELA | | INNER CITY | | 20546 | | 20813 | | 21068 | | 21312 | | 21540 | | 21773 | | 21971 | | 22182 | | 22376 | | 22562 | |
| 2709152 | | TRAIPU | | INNER CITY | | 12655 | | 12775 | | 12889 | | 12993 | | 13089 | | 13200 | | 13465 | | 13527 | | 13601 | | 13668 | |
| 2709202 | | UNIÃO DOS PALMARES | | INNER CITY | | 32058 | | 32314 | | 32564 | | 32809 | | 33046 | | 33227 | | 33409 | | 33577 | | 33783 | | 33955 | |
| 2709301 | | VIÇOSA | | INNER CITY | | 13402 | | 13384 | | 13366 | | 13351 | | 13339 | | 13330 | | 13324 | | 13324 | | 13308 | | 13295 | |
