# Supplementary material for: Spatial-temporal patterns of homicide in socioeconomically deprived settings: violence in Alagoas, Brazil, 2006‒2015
Source: Glob Health Action. 2021 Aug 2;14(1):1952752. doi: 10.1080/16549716.2021.1952752 (PMC8330714; doi:10.1080/16549716.2021.1952752)
Supplement: Supplemental Material [file ZGHA_A_1952752_SM0888.zip › Supplementary/Supplementary file_2.docx]

| Supplementary material 2. List of cities according to economic status and homicide rate (2009-2011). | | | | |
| --- | --- | --- | --- | --- |
| **CENSUS CODE** | **CITY** | **REGION** | **MONTHLY INCOME* (R$**)** | **HOMICIDE RATE (2009-2011)** |
| 2700102 | ÁGUA BRANCA | INNER CITY | 508.52 | 26.6 |
| 2700201 | ANADIA | INNER CITY | 530.14 | 24.0 |
| 2700300 | ARAPIRACA | INNER CITY | **780.64 (5th)** | 73.4 |
| 2700409 | ATALAIA | METRO REGION | 528.56 | 56.6 |
| 2700508 | BARRA DE SANTO ANTÔNIO | METRO REGION | 564.57 | 36.2 |
| 2700607 | BARRA DE SÃO MIGUEL | METRO REGION | **786.42 (4th)** | **85.0 (6th)** |
| 2700706 | BATALHA | INNER CITY | 517.76 | 26.4 |
| 2700805 | BELÉM | INNER CITY | 590.84 | 56.6 |
| 2700904 | BELO MONTE | INNER CITY | 501.29 | 32.0 |
| 2701001 | BOCA DA MATA | INNER CITY | 566.98 | 62.4 |
| 2701100 | BRANQUINHA | INNER CITY | 475.80 | 73.0 |
| 2701209 | CACIMBINHAS | INNER CITY | 535.96 | 50.5 |
| 2701308 | CAJUEIRO | INNER CITY | 515.83 | 47.3 |
| 2701357 | CAMPESTRE | INNER CITY | 540.33 | 4.9 |
| 2701407 | CAMPO ALEGRE | INNER CITY | 572.08 | 22.2 |
| 2701506 | CAMPO GRANDE | INNER CITY | 441.22 | 46.3 |
| 2701605 | CANAPI | INNER CITY | 417.52 | 41.0 |
| 2701704 | CAPELA | INNER CITY | 528.71 | 52.8 |
| 2701803 | CARNEIROS | INNER CITY | 442.69 | 46.6 |
| 2701902 | CHÃ PRETA | INNER CITY | 487.50 | 4.5 |
| 2702009 | COITÉ DO NÓIA | INNER CITY | 437.88 | 38.3 |
| 2702108 | COLÔNIA LEOPOLDINA | INNER CITY | 528.86 | 41.8 |
| 2702207 | COQUEIRO SECO | METRO REGION | 593.51 | **75.7 (9th)** |
| 2702306 | CORURIPE | INNER CITY | 650.13 | **74.7 (10th)** |
| 2702355 | CRAÍBAS | INNER CITY | 425.29 | 41.2 |
| 2702405 | DELMIRO GOUVEIA | INNER CITY | **663.23 (10th)** | 47.5 |
| 2702504 | DOIS RIACHOS | INNER CITY | 438.11 | 26.6 |
| 2702553 | ESTRELA DE ALAGOAS | INNER CITY | 401.67 | 26.1 |
| 2702603 | FEIRA GRANDE | INNER CITY | 443.68 | 57.4 |
| 2702702 | FELIZ DESERTO | INNER CITY | 480.16 | 37.0 |
| 2702801 | FLEXEIRAS | INNER CITY | 474.27 | 57.5 |
| 2702900 | GIRAU DO PONCIANO | INNER CITY | 445.79 | 36.1 |
| 2703007 | IBATEGUARA | INNER CITY | 448.07 | 48.9 |
| 2703106 | IGACI | INNER CITY | 516.67 | 38.3 |
| 2703205 | IGREJA NOVA | INNER CITY | 605.18 | 27.6 |
| 2703304 | INHAPI | INNER CITY | 494.13 | 25.2 |
| 2703403 | JACARÉ DOS HOMENS | INNER CITY | 491.28 | 47.6 |
| 2703502 | JACUÍPE | INNER CITY | 484.43 | 36.8 |
| 2703601 | JAPARATINGA | INNER CITY | 605.45 | 24.9 |
| 2703700 | JARAMATAIA | INNER CITY | 449.27 | 23.2 |
| 2703759 | JEQUIÁ DA PRAIA | INNER CITY | 454.24 | 24.1 |
| 2703809 | JOAQUIM GOMES | INNER CITY | 434.79 | 64.2 |
| 2703908 | JUNDIÁ | INNER CITY | 576.14 | 7.7 |
| 2704005 | JUNQUEIRO | INNER CITY | 620.70 | 41.9 |
| 2704104 | LAGOA DA CANOA | INNER CITY | 493.21 | 35.3 |
| 2704203 | LIMOEIRO DE ANADIA | INNER CITY | 524.76 | 21.5 |
| 2704302 | MACEIÓ (Capital) | METRO REGION | **1377.07 (1st)** | **91.2 (4th)** |
| 2704401 | MAJOR ISIDORO | INNER CITY | 527.86 | 30.7 |
| 2704906 | MAR VERMELHO | INNER CITY | 456.66 | 8.8 |
| 2704500 | MARAGOGI | INNER CITY | 552.45 | 34.7 |
| 2704609 | MARAVILHA | INNER CITY | 490.21 | 25.0 |
| 2704708 | MARECHAL DEODORO | METRO REGION | **854.30 (2nd)** | **98.0 (3rd)** |
| 2704807 | MARIBONDO | INNER CITY | 543.50 | 54.3 |
| 2705002 | MATA GRANDE | INNER CITY | 440.93 | 28.7 |
| 2705101 | MATRIZ DE CAMARAGIBE | INNER CITY | 515.07 | 44.7 |
| 2705200 | MESSIAS | METRO REGION | 578.35 | **88.3 (5th)** |
| 2705309 | MINADOR DO NEGRÃO | INNER CITY | 479.16 | 30.5 |
| 2705408 | MONTEIRÓPOLIS | INNER CITY | 397.77 | 13.9 |
| 2705507 | MURICI | METRO REGION | 512.70 | 49.4 |
| 2705606 | NOVO LINO | INNER CITY | 527.75 | 10.7 |
| 2705705 | OLHO D'ÁGUA DAS FLORES | INNER CITY | 537.15 | 41.1 |
| 2705804 | OLHO D'ÁGUA DO CASADO | INNER CITY | 485.33 | 22.7 |
| 2705903 | OLHO D'ÁGUA GRANDE | INNER CITY | 397.05 | 0.0 |
| 2705903 | OLIVENÇA | INNER CITY | 440.19 | 29.1 |
| 2706000 | OURO BRANCO | INNER CITY | 478.05 | 8.9 |
| 2706109 | PALESTINA | INNER CITY | 476.91 | 6.3 |
| 2706109 | PALMEIRA DOS ÍNDIOS | INNER CITY | **749.30 (7th)** | 54.0 |
| 2706307 | PÃO DE AÇÚCAR | INNER CITY | 502.13 | 29.7 |
| 2706406 | PARICONHA | INNER CITY | 480.99 | 18.8 |
| 2706422 | PARIPUEIRA | METRO REGION | 654.63 | 53.9 |
| 2706448 | PASSO DE CAMARAGIBE | INNER CITY | 456.01 | 32.7 |
| 2706505 | PAULO JACINTO | INNER CITY | 487.99 | 73.7 |
| 2706604 | PENEDO | INNER CITY | **672.76 (9th)** | 67.7 |
| 2706703 | PIAÇABUÇU | INNER CITY | 467.87 | 56.1 |
| 2706802 | PILAR | METRO REGION | 632.93 | **109.2 (1st)** |
| 2706901 | PINDOBA | INNER CITY | 595.95 | 11.2 |
| 2706901 | PIRANHAS | INNER CITY | 608.00 | 54.5 |
| 2707107 | POÇO DAS TRINCHEIRAS | INNER CITY | 457.14 | 32.5 |
| 2707206 | PORTO CALVO | INNER CITY | 543.25 | 26.3 |
| 2707305 | PORTO DE PEDRAS | INNER CITY | 496.05 | 7.6 |
| 2707404 | PORTO REAL DO COLÉGIO | INNER CITY | 487.39 | 38.3 |
| 2707503 | QUEBRANGULO | INNER CITY | 483.37 | 44.8 |
| 2707602 | RIO LARGO | METRO REGION | **713.81 (8th)** | 69.1 |
| 2707701 | ROTEIRO | INNER CITY | 467.36 | 38.7 |
| 2707800 | SANTA LUZIA DO NORTE | METRO REGION | 614.04 | 37.4 |
| 2707909 | SANTANA DO IPANEMA | INNER CITY | 625.14 | 57.3 |
| 2708006 | SANTANA DO MUNDAÚ | INNER CITY | 431.00 | 14.7 |
| 2708105 | SÃO BRÁS | INNER CITY | 502.51 | 24.0 |
| 2708204 | SÃO JOSÉ DA LAJE | INNER CITY | 604.55 | 72.4 |
| 2708303 | SÃO JOSÉ DA TAPERA | INNER CITY | 464.85 | 39.6 |
| 2708402 | SÃO LUÍS DO QUITUNDE | INNER CITY | 565.35 | 43.7 |
| 2708501 | SÃO MIGUEL DOS CAMPOS | INNER CITY | **759.89 (6th)** | **102.1 (2nd)** |
| 2708600 | SÃO MIGUEL DOS MILAGRES | INNER CITY | 506.32 | 36.0 |
| 2708709 | SÃO SEBASTIÃO | INNER CITY | 485.19 | 71.4 |
| 2708808 | SATUBA | METRO REGION | **852.28 (3nd)** | **79.4 (8th)** |
| 2708907 | SENADOR RUI PALMEIRA | INNER CITY | 377.95 | 22.2 |
| 2708956 | TANQUE D'ARCA | INNER CITY | 428.54 | 26.3 |
| 2709004 | TAQUARANA | INNER CITY | 438.54 | 57.5 |
| 2709103 | TEOTÔNIO VILELA | INNER CITY | 548.85 | **81.4 (7th)** |
| 2709152 | TRAIPU | INNER CITY | 461.80 | 37.6 |
| 2709202 | UNIÃO DOS PALMARES | INNER CITY | 616.26 | 71.2 |
| 2709301 | VIÇOSA | INNER CITY | 555.39 | 44.3 |
| *Considering the 2010 Brazilian Census. **R$ = Brazilian currency. In 2010, 1 $ (American dollar) was approximately 1.86 R$. In Bold the top 10 wealthiest cities. | | | | |
